# Supplementary material for: Anthocyanins Formulated with Carboxymethyl Starch for Gastric and Intestinal Delivery
Source: Molecules. 2022 Oct 26;27(21):7271. doi: 10.3390/molecules27217271 (PMC9657027; doi:10.3390/molecules27217271)
Supplement: Supplementary file 1 [file molecules-27-07271-s001.zip › molecules-1982911-supplementary.pdf]

SUPPLEMENTARY MATERIAL

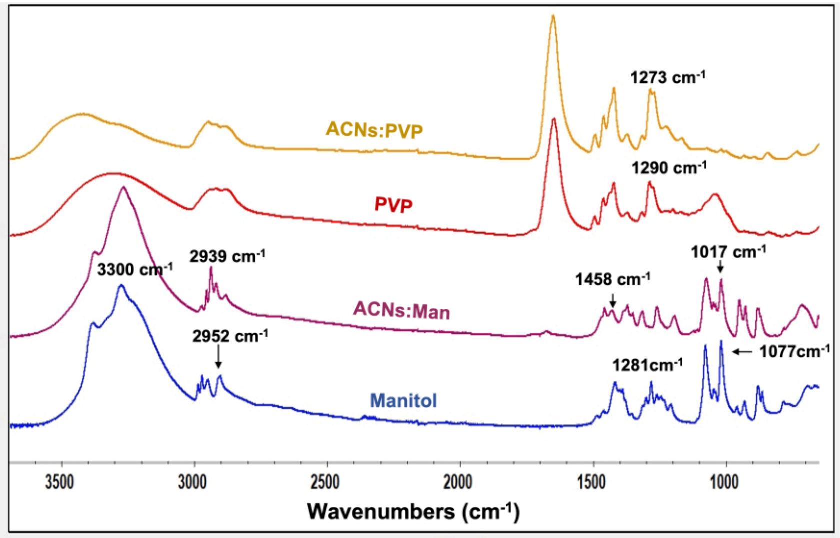

Figure S1. FTIR spectra of Mannitol, Anthocyanins:Mannitol (ACNs:Man), Polyvinylpyrrolidone (PVP) and Anthocyanins:Polyvinylpyrrolidone (ACNs:PVP).

The mannitol spectrum exhibited a strong signal at 3300 cm^{-1} related to the -OH stretching in hydrogen bonds. The signal at 2952 cm^{-1} was attributed C-H vibrations and the bands 1281.1 and 1077 cm^{-1} belonged to C-O and C-C stretching, respectively. In Anthocyanins:Mannitol (ACNs:Man) spectrum (Figure S1) the hydrogen group increased its intensity at 3300 cm^{-1} . The C-H signal shifted to 2939 cm^{-1}, while the peaks related to C=C and C-H stretching became sharper, less intense and with new signals (1458 and 1017.7 cm^{-1}, respectively). In PVP, the sign of the C-H group shifted to 2947 cm^{-1}, while the C=O overlapped at the band at 1646 cm^{-1} due to its interaction with C=O groups of ACNs. In addition, the PVP vibration signature at 1290 cm^{-1}, related C-N vibrations, became 1273.2 cm^{-1} in Anthocyanins:Polyvinylpyrrolidone (ACNs:PVP) spectrum possibly due to its interaction with ACNs pyran rings [39,40].

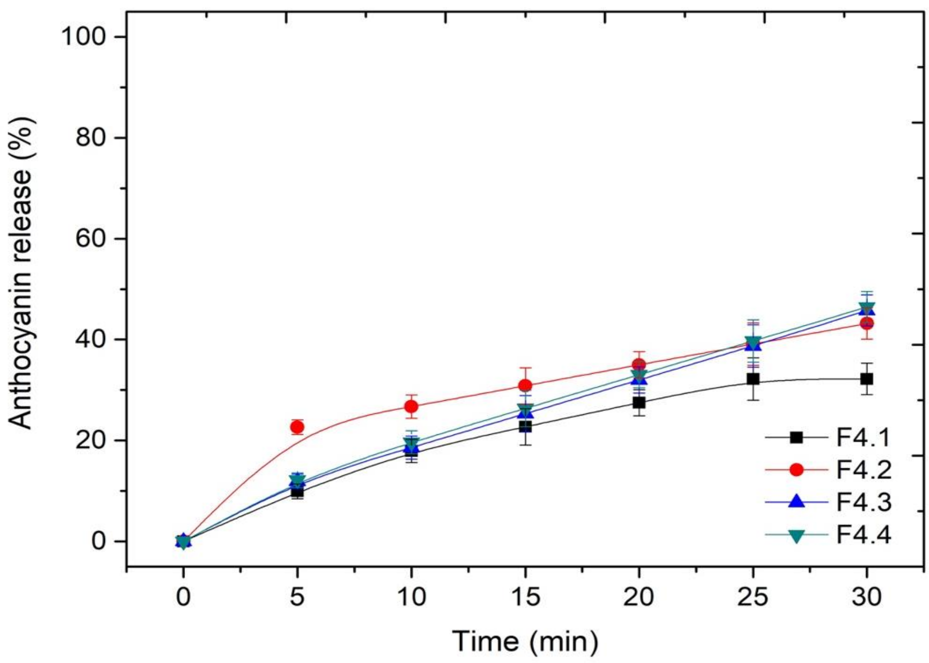

Figure S2. Dissolution profiles of ACNs formulations for immediate gastric release (F4.1, F4.2, F4.3 and F.4.4, 30 min/SGF)

Table S1. Detailed composition of the monolithic tablets.

Sample	Composition (% w/w)									
	CMS	ACNs	ACNs:Man	ACNs:PVP	ACNs:CMS*	CCS	Prosolv•	Gum arabic	Explotab•	MCC
F1	50	50	-	-	-	-	-	-	-	-
F2	50	-	50	-	-	-	-	-	-	-
F3	50	-	-	50	-	-	-	-	-	
F4	-	-	-	-	100	-	-	-	-	-
F4.1	-	-	-	-	90	10	-	-	-	-
F4.2	-	-	-	-	67	6	27	-	-	-
F4.3	-	-	-	-	67	6	-	27	-	-
F4.4	-	-	-	-	47	-	-	35	18	-
F4.5	-	-	-	-	47	-	-	-	18	35
F4.6	-	-	-	-	47	18	-	-	-	35
F4.7	-	-	-	-	55	10	-	-	-	35
F4.8	-	-	-	-	80	10	-	-	-	10

CMS - Carboxymethyl starch; ACNs - Anthocyanin dried extract; ACNs:Man -Anthocyanins:Mannitol powder; ACNs:PVP - Anthocyanins:Polyvinylpyrrolidone powder; CCS - Crosscarmellose sodium; MCC - Microcrystalline cellulose; *ACNs:CMS: Complex obtained from the mixture and freeze-drying of CMS with ACNs.
